# Severe hypertriglyceridemia in a subject with disturbed life style and poor glycemic control without recurrence of acute pancreatitis: a case report

**DOI:** 10.1186/s12902-019-0425-9

**Published:** 2019-08-30

**Authors:** Shintaro Irie, Takatoshi Anno, Fumiko Kawasaki, Ryo Shigemoto, Shuhei Nakanishi, Kohei Kaku, Hideaki Kaneto

**Affiliations:** 1 Department of General Internal Medicine 1, Kawasaki Medical School, 2-6-1 Nakasange, Kita-ku, Okayama, 700-8505 Japan; 2 Department of Diabetes, Metabolism and Endocrinology, Kawasaki Medical School, Kurashiki, 701-0192 Japan

**Keywords:** Severe hypertriglyceridemia, Insulin effect, Poor glycemic control, After pancreatitis

## Abstract

**Background:**

Hypertriglyceridemia is often observed as the result of lipid abnormality and frequently associated with other lipid and metabolic disorders. Aggravation of hypertriglyceridemia is caused by various conditions. However, severe hypertriglyceridemia is usually induced by an addition of some secondary clinical conditions such as uncontrolled type 2 diabetes mellitus (T2DM) and obesity with insulin resistance.

**Case presentation:**

A 40-year-old man with 4-year history of dyslipidemia and T2DM visited after his interruption of therapy for about 1.5 years. His past history was acute pancreatitis. His life style was markedly disturbed, and he had a lot of risk factors for hypertriglyceridemia. Surprisingly, his serum triglyceride level was as high as 16,900 mg/dL. His aggravation and remission of hypertriglyceridemia were closely associated with the alteration of RLP-cholesterol levels in dyslipidemia and glycoalbumin and ketone body levels in T2DM.

**Conclusion:**

We report very severe hypertriglyceridemia, which seemed to be caused by markedly disturbed life style and poorly controlled T2DM. Total therapy with diet and drug for each disease is very important for the improvement of very severe hypertriglyceridemia. This case report suggests that very severe hypertriglyceridemia alone does not necessarily bring out acute pancreatitis, although it is very important to check pancreatitis markers in such a situation.

## Background

Hypertriglyceridemia is often observed as the result of lipid abnormality and frequently associated with other lipid and metabolic disorders. In addition, hypertriglyceridemia results from overproduction of triglyceride-rich lipoproteins. The metabolisms of triglyceride-rich lipoproteins are promoted by lipoprotein lipase (LPL) and LPL activity is enhanced by insulin effect [1].

Hypertriglyceridemia is a common clinical diagnosis and sometimes classified as primary or secondary one [2]. Aggravation of hypertriglyceridemia is caused by various conditions. However, severe hypertriglyceridemia is usually induced by an addition of some secondary clinical conditions such as uncontrolled type 2 diabetes mellitus (T2DM) and obesity with insulin resistance.

Severe hypertriglyceridemia warrants treatment because it could lead to an increased risk of acute pancreatitis. We should bear in mind that over 2000 mg/dL of triglyceride is a high risk for pancreatitis [3].

## Case presentation

A 40-year-old man with 4-year history of dyslipidemia and T2DM had a symptom of general fatigue. He enjoyed SUMOU, which is a traditional Japanese sport, in his high school days, and thereby he ate over 10,000 kcal/day of diet every day when he was young. For that reason, he sometimes ate 3000–5000 kcal of diet at once even at that time. His past history was acute pancreatitis at the age of 36, and after the onset of pancreatitis, he stopped drinking alcohol and smoking.

At the age of 36, metabolic markers were as follows: total cholesterol, 644 mg/dL; Low Density Lipoprotein (LDL)-cholesterol, 127 mg/dL; High Density Lipoprotein (HDL)-cholesterol, 23 mg/dL; triglyceride, 3207 mg/dL; plasma glucose, 244 mg/dL; hemoglobin A1c (HbA1c), 10.3%. His height, body weight and BMI were 180.0 cm, 121.5 kg and 37.5 kg/m^2^, respectively. Acute pancreatitis markers were as follows: lipase, 987 U/L; trypsin, 6662 ng/mL; pancreatic phospholipase A2, 1150 ng; pancreatic amylase, 290 IU/L. He had no family history of them. Although during hospitalization period we treated him with intensive insulin therapy for T2DM, after the improvement of acute pancreatitis, he was taking 400 mg/day of bezafibrate and 1800 mg/day of ethyl icosapentate for the treatment of dyslipidemia and 30 mg/day of mitiglinide, 0.6 mg/day of voglibose, 1500 mg/day of metformin and insulin therapy (4 units of glargine) for T2DM at discharge. His triglyceride levels were 64–734 mg/dL for 2 years after discharge of acute pancreatitis, but he stopped the medication for dyslipidemia on his own judgement. We started 10 mg/day of ezetimibe for the treatment of dyslipidemia because his triglyceride level was as high as 1921 mg/dL, and after then his triglyceride level was decreased to 416 mg/dL. His HbA1c levels were 7.0–9.1% for 2 years after discharge in outpatient clinic in spite of stopping insulin. At that time, he was treated with mitiglinide, 0.6 mg/day of voglibose, 1500 mg/day of metformin and 0.9 mg/day of liraglutide, but he stopped the medication for T2DM on his own judgement. We started the same medication and added 2.5 mg/day of luseogliflozin for the treatment of T2DM because his HbA1c levels were as high as 12.9%. However, after 6 months, he stopped receiving the treatment for about 1.5 years on his own judgement. In outpatient clinic, his body weight was 111.6–120.0 kg.

**Fig. 1:**
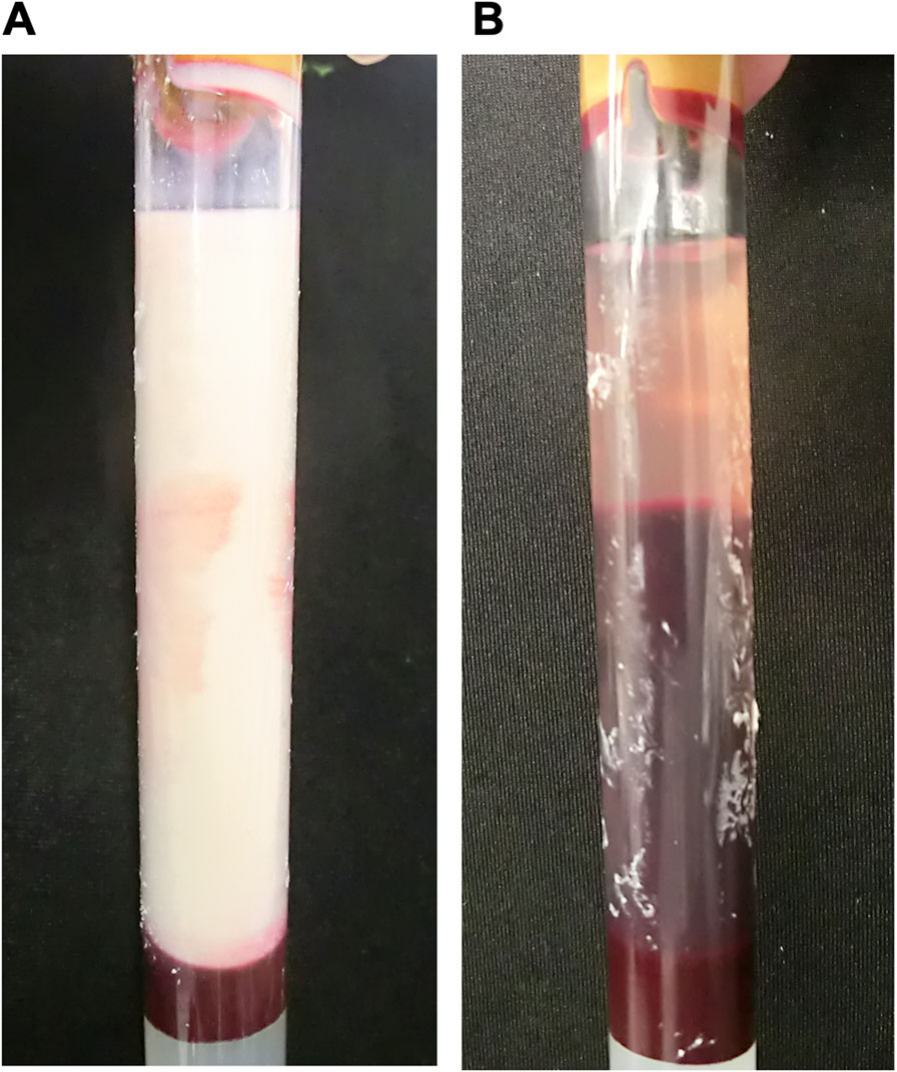
**a**. Blood sample drawn in an emergency room showed white and opaque fluid. **b**. Blood sample drawn from the same patient 3 months later showed clear fluid

At the age of 40, after his interruption of therapy, he felt general fatigue and visited the emergency room of our office once more. His height, body weight and BMI were 180.0 cm, 114.4 kg and 35.3 kg/m^2^, respectively. His vital signs were normal. Laboratory data were as follows: plasma glucose, 260 mg/dL; plasma insulin, 27.6 μU/mL; HbA1c, 12.9%; glycoalbumin 33.5%; total cholesterol, 1055 mg/dL; LDL-cholesterol, 240 mg/dL; HDL-cholesterol, 17 mg/dL; triglyceride, 11,175 mg/dL. Urine glucose (3+) and urine ketone body (2+) were detected in urinalysis. Furthermore, his blood sample showed white and opaque (Fig. 1a). He did not agree to the hospitalization and frequent insulin injection. In addition, his dyslipidemia and T2DM had been improved with previous oral medication. Therefore, we started 160 mg/day of fenofibrate and insulin therapy (8 units of degludec) on an outpatient basis. At that time, his life style was markedly disturbed; for example, he drunk over 1.5 L of PET bottle of juice, ate a lot of snacks and fruits and fast food in eating out. He sometimes continued to eat 3000–5000 kcal of diet at once even at that time. We treated him with therapeutic lifestyle changes and diet therapy of 2200 kcal/day (about 30 kcal/ideal body weight kg). After 4 days, we examined laboratory data in the fasting state. Laboratory data were as follows (Table 1): total cholesterol, 705 mg/dL; LDL-cholesterol, 117 mg/dL; HDL-cholesterol, 20 mg/dL; triglyceride, 5555 mg/dL; Remnant like particle (RLP)-cholesterol, 264.8 mg/dL; Apolipoprotein A-I, 121 mg/dl; Apolipoprotein B, 145 mg/dl; Apolipoprotein E, 33.4 mg/dl. In addition, lipoprotein fractions showed Very Low Density Lipoprotein (VLDL) and MIDBAND fractions were elevated (VLDL, 44%; MIDBAND, 33%). Another fasting laboratory data were as follows: plasma glucose, 279 mg/dL, total ketone bodies, 1275.1 μmol/L; β-hydroxybutyrate, 750.8 μmol/L; acetoacetate 524.3 μmol/L. TSH, FT3 and FT4 levels were 1.562 μIU/mL, 2.83 pg/mL and 1.29 ng/dL. In spite of such very severe hypertriglyceridemia, he did not have acute pancreatitis (lipase, 30 U/L; trypsin, 262 ng/mL; pancreatic phospholipase A2, 167 ng/dL; pancreatic amylase, 15 IU/L). He frequently visited our office every 1–2 weeks, and we increased insulin therapy little by little (from 8 to 14 units of degludec) in outpatient clinic. One month later, his serum triglyceride level was reduced.

**Table 1:** Time course of various dyslipidemia and diabetes markers before and after therapy

Dyslipidemia marker	in an emergency room	after 4 days	after 1 month	after interruption of therapy	1 month after treatment restart	1.5 months after treatment restart	Reference range
Total cholesterol (mg/dL)	1055	705	233	1368	131	224	142–248
LDL cholesterol (mg/dL)	240	117	96	490	78	151	65–139
HDL cholesterol (mg/dL)	17	20	34	11	26	31	40–90
Triglyceride (mg/dL)	11,175	5555	675	16,900	298	328	40–149
RLP- cholesterol (mg/dL)	N/A	264.8	N/A	414.9	N/A	15.8	0.0–7.5
Apolipoprotein A-I (mg/dL)		121		N/A		84	119–155
Apolipoprotein A-II (mg/dL)		N/A		N/A		15.1	25.9–35.7
Apolipoprotein B (mg/dL)		145		N/A		140	73–109
Apolipoprotein C-II (mg/dL)		N/A		N/A		9.1	1.8–4.6
Apolipoprotein C-III (mg/dL)		N/A		N/A		11.8	5.8–10.0
Apolipoprotein E (mg/dL)		33.4		N/A		7.0	2.7–4.3
lipoprotein fractions							
VLDL (%)		44		52		16	3–19
MIDBAND (%)		33		37		24	
LDL (%)		9		6		46	46–68
HDL (%)		11		5		14	22–74
BAND (%)		3		–		–	
pre-heparin LPL (ng/mL)		N/A		N/A		35	
Diabetes marker	in an emergency room	after 4 days	after 1 month	after interruption of therapy	1 month after treatment restart	1.5 months after treatment restart	Reference range
Plasma Glucose (mg/dL)	260	N/A	277	310	115	110	73–109
Glycoalbumin (%)	33.5	N/A	25.0	27.8	23.2	21.6	12.4–16.3
Total ketone bodies (μmol/L)	N/A	1275.1	88.6	3870.0	64.8	648.0	0.0–130.0
Acetoacetate (μmol/L)	N/A	524.3	46.8	1610.0	30.9	216.9	0.0–55.0
Beta-hydroxybutylate (μmol/L)	N/A	750.8	41.8	2260.0	33.9	431.1	0.0–85.0

Abbreviation: *LDL* Low-density lipoproteins, *HDL* High-density lipoprotein, *RLP* remnant like particle, *LPL* lipoprotein lipase

*N/A* not applicable

Two months later, however, his life style became markedly disturbed once more and quitted his therapy again. At that time, surprisingly, his serum triglyceride was markedly increased up to 16,900 mg/dL (Table 1). He ate high calorie diet including large amounts of rice, which is known as a main source of carbohydrate in Japan. It was estimated that he ate over 1000 kcal of rice. In addition, he ate large amounts of oily food such as fried chicken and fried potato. He failed to precisely explain how many amounts of fat he ate, but it was estimated that he ate 3000–5000 kcal of diet in total at once. Moreover, he drunk over 2.0 L of PET bottle of juice and ate a lot of snacks at the same time. He stopped injecting insulin and his glycemic control was very poor (plasma glucose, 310 mg/dL; HbA1c, 13.1%). We re-tried the education, diet therapy and medication. One month later, serum triglyceride level was dramatically decreased which was accompanied again by the amelioration of glycemic control and marked reduction of ketone body (Table 1). Furthermore, his blood sample showed clear fluid (Fig. 1b). At that time, his medication was 160 mg/day of fenofibrate for the treatment of dyslipidemia and insulin therapy (18 units of glargine) for T2DM. We examined lipid parameters once more 1.5 months after treatment restart. Laboratory data were as follows (Table 1): total cholesterol, 224 mg/dL; LDL-cholesterol, 151 mg/dL; HDL-cholesterol, 31 mg/dL; triglyceride, 328 mg/dL; RLP-cholesterol, 15.8 mg/dL; Apolipoprotein A-I, 84 mg/dl; Apolipoprotein A-II, 15.1 mg/dl; Apolipoprotein B, 140 mg/dl; Apolipoprotein C-II, 91 mg/dl; Apolipoprotein C-III, 11.8 mg/dl; Apolipoprotein E, 7.0 mg/dl. Lipoprotein fractions showed VLDL and MIDBAND fractions were decreased under stable dyslipidemia (VLDL, 16%; MIDBAND, 24%). After then, his body weight was reduced to under 110 kg.

## Discussion and conclusions

Herein we report very severe hypertriglyceridemia, which seemed to be caused by disturbed life style and poorly controlled T2DM. Granted that he had a lot of risk factors for hypertriglyceridemia, his serum triglyceride level was as high as 16,900 mg/dL. This value is extremely high, considering that over 2000 mg/dL of triglyceride is a high risk for pancreatitis. Furthermore, to the best of our knowledge, this triglyceride level in this subject is the highest in medical literature [4].

Insulin resistance is caused by metabolic syndrome and uncontrolled T2DM especially in obese subjects. This patient had very severe obesity among Japanese subjects. Since it is known that pancreatic beta-cell function in Japanese is genetically weak compared to Caucasians [5]. We assume that not only dietary lipids and carbohydrates but also free fatty acids secreted from adipocytes could be involved in pancreatic beta-cell dysfunction. Such phenomena are well known as lipotoxicity [6]. In addition, it is known that decreased insulin effect leads to the reduction of LPL activity. Since activated LPL induces metabolisms of triglyceride-rich lipoprotein, decreased insulin effect leads to the reduction of LPL activity and the elevation of triglyceride-rich lipoprotein [1]. His pre-heparin LPL levels was 35 ng/mL (Reference range 45–63 ng/mL). Surprisingly, his triglyceride levels were elevated to over 10,000 mg/dL at least twice. At that time, his life style was markedly disturbed with high calorie diet including large amounts of fat and carbohydrate and he had very severe obesity among Japanese subjects. In addition, it seemed that his severe hypertriglyceridemia was induced by obesity together with insulin resistance. We assume that not only dietary lipids and carbohydrates but also free fatty acids secreted from adipocytes could be involved in very severe hypertriglyceridemia in this subject. Moreover, his severe hypertriglyceridemia was aggravated by uncontrolled T2DM. At that time, he suffered from ketosis and his insulin effect was not enough at all. On the other hand, his severe hypertriglyceridemia was improved by diet therapy with low lipid and carbohydrate and medication with fibrate and insulin. These data showed the mechanism of severe hypertriglyceridemia induced by poorly controlled T2DM. In addition, when triglyceride levels were elevated to over 10,000 mg/dL, RLP-cholesterol, which is known to stagnate the metabolism of triglyceride, was very much increased. RLP-cholesterol is elevated in hypertriglyceridemia patients with metabolic syndrome and T2DM [7]. And increased remnant lipoprotein is an abnormal concentration of triglyceride, and lipoprotein fractions show the elevation of VLDL and MIDBAND [8].

It is important to know that very severe hypertriglyceridemia is a risk for acute pancreatitis. In general, it is thought that over 2000 mg/dL of triglyceride levels is a high risk for pancreatitis [3]. However, his pancreatitis markers were within normal range. One possibility is that he stopped drinking alcohol and thereby he might not have repeated acute pancreatitis in spite of very severe hypertriglyceridemia.

There is a limitation in this case report. His genetic background and laboratory data for primary hyperlipidaemia such as familial hyperlipidaemia were not clear. In general, in order to perform its definite diagnosis, it is necessary to examine several points such as the Apolipoprotein activity, phenotype of Apolipoprotein and LPL activity. First, he had no family history of hyperlipidaemia. Second, there were no findings in physical examination such as xanthelasma palpebrarum, tuberous xanthoma and plane xanthoma and atherosclerosis in computed tomography, magnetic resonance imaging and echocardiography. In addition, although he had elevated VLDL and MIDBAND fractions in lipoprotein fractions under severe hypertriglyceridemia, VLDL and MIDBAND fractions were decreased under stable dyslipidemia. Moreover, his various Apolipoprotein levels were elevated but were not decreased under stable dyslipidemia. His pre-heparin LPL levels were slightly decreased under stable dyslipidemia, while it was reported that pre-heparin LPL levels were suppressed under metabolic syndrome and T2DM [1, 9]. However, since we did not examine the basal and post-heparin LPL activities before insulin treatment, we failed to show the improvement of LPL activities by insulin treatment. Therefore, although we think that very severe hypertriglyceridemia in this subject was induced by markedly disturbed life style and poorly controlled type 2 diabetes mellitus, we cannot exclude the possibility that he had primary hyperlipidaemia such as familial hyperlipidaemia. In addition, in general ketonemia is recovered after obtaining good glycemic control. However, this patient had ketonemia even after obtaining relatively good glycemic control (1.5 months after treatment restart in Table 1). Although speculative, such ketonemia might be, at least in part, related with some lifestyle habit such meal variation. However, it is very difficult to explain the real reason and/or its precise mechanism.

Taken together, we should bear in mind that disturbed life style and poorly controlled T2DM lead to very severe hypertriglyceridemia. And total care for T2DM and hypertriglyceridemia is very important for the improvement of very severe hypertriglyceridemia. In addition, this case report suggests that very severe hypertriglyceridemia alone does not necessarily bring out acute pancreatitis, although, needless to say, it is very important to check pancreatitis markers in such a situation.

## Data Availability

Not applicable.

## Abbreviations

HbA1c: hemoglobin A1c
HDL: High Density Lipoprotein
LDL: Low Density Lipoprotein
LPL: lipoprotein lipase
RLP: Remnant like particle
T2DM: type 2 diabetes mellitus
VLDL: Very Low Density Lipoprotein
